# Granulocyte-macrophage colony-stimulating factor is a key mediator in experimental osteoarthritis pain and disease development

**DOI:** 10.1186/ar4037

**Published:** 2012-09-20

**Authors:** Andrew D Cook, Jarrad Pobjoy, Stefan Steidl, Manuela Dürr, Emma L Braine, Amanda L Turner, Derek C Lacey, John A Hamilton

**Affiliations:** 1 Arthritis and Inflammation Research Centre, Department of Medicine, The University of Melbourne, Victoria, 3010, Australia; 2 MorphoSys AG, Lena-Christ-Strasse 48, Martinsried/Planegg, 82152, Germany

## Abstract

**Introduction:**

Granulocyte-macrophage colony-stimulating factor (GM-CSF) has been shown to be important in the development of inflammatory models of rheumatoid arthritis and there is encouraging data that its blockade may have clinical relevance in patients with rheumatoid arthritis. The aims of the current study were to determine whether GM-CSF may also be important for disease and pain development in a model of osteoarthritis.

**Methods:**

The role of GM-CSF was investigated using the collagenase-induced instability model of osteoarthritis. We studied both GM-CSF-/- mice and wild-type (C57BL/6) mice treated prophylactically or therapeutically with a monoclonal antibody to GM-CSF. Disease development (both early and late) was evaluated by histology and knee pain development was measured by assessment of weight distribution.

**Results:**

In the absence of GM-CSF, there was less synovitis and matrix metalloproteinase-mediated neoepitope expression at week 2 post disease induction, and less cartilage damage at week 6. GM-CSF was absolutely required for pain development. Therapeutic neutralization of GM-CSF not only abolished the pain within 3 days but also led to significantly reduced cartilage damage.

**Conclusions:**

GM-CSF is key to the development of experimental osteoarthritis and its associated pain. Importantly, GM-CSF neutralization by a therapeutic monoclonal antibody-based protocol rapidly and completely abolished existing arthritic pain and suppressed the degree of arthritis development. Our results suggest that it would be worth exploring the importance of GM-CSF for pain and disease in other osteoarthritis models and perhaps clinically for this form of arthritis.

## Introduction

Granulocyte-macrophage colony-stimulating factor (GM-CSF) was originally defined as a hemopoietic growth factor [1]. However, it can act on mature myeloid cells [2] and it has other functions, acting as a proinflammatory cytokine [2-5] and in dendritic cell function [6]. More specifically, its depletion can have profound effects on disease severity and progression in many inflammatory arthritis models [7-10]; encouragingly, initial results suggest that antibody blockade of the GM-CSF receptor has therapeutic benefit in rheumatoid arthritis (RA) [11,12].

Osteoarthritis (OA) is the most common rheumatic disorder. The pathogenic characteristics of OA are loss of cartilage with associated underlying bony changes consisting of sclerosis, subchondral bone collapse, bone cysts, and osteophyte formation [13]. Pain is one of the most important symptoms of OA as it causes a significant impairment in function. The etiology of OA is likely to be multifactorial, with mechanical, metabolic and inflammatory contributions. Recent histologic evidence indicates that synovitis can be an early feature in OA, even in joints where it could not be detected clinically [14-16], with a mixed inflammatory infiltrate consisting mainly of macrophages and with proinflammatory mediator production (for example, TNF, IL-1β) [17,18]. It has been argued that OA synovial inflammation is qualitatively similar to that in RA, differing only in magnitude [19].

The collagenase-induced OA model is based on the induction of joint instability by intra-articular injection of collagenase. This causes weakening of the ligaments, leading to an OA-like pathology, including cartilage matrix erosion and osteophyte formation within 6 weeks [20,21]. It is macrophage-dependent; the macrophages mediate osteophyte formation and fibrosis in the early stages [21,22]. Given that the major functions of GM-CSF appear to be as a pro-survival and 'activating' factor for myeloid cells [2], in the current study we investigated whether this experimental OA model is dependent on GM-CSF. As pain is an important symptom of OA, with a complex relationship with tissue damage [23], the requirement of GM-CSF for development of such pain in the collagenase-induced arthritis model was also sought. We have recently shown that GM-CSF is key to the development of arthritic pain in a number of inflammatory arthritis models [24].

We report here that GM-CSF is an important mediator in the progression of both the pain and disease in this OA-like model.

## Methods

### Mice

GM-CSF gene-deficient (GM-CSF-/-) mice were backcrossed onto the C57BL/6 background for 12 generations [8,25,26]. Mice of both sexes, 8 to 12 weeks of age, were used in all experiments. Mice were housed five per cage and the male:female distribution was comparable for all experimental groups. All experiments were approved by The University of Melbourne Animal Ethics Committee.

### Collagenase-induced arthritis

Arthritis was induced as published [21]. Briefly, mice received an intra-articular injection of one unit of collagenase type VII (Sigma-Aldrich, St. Louis, Missouri, USA) on days 0 and 2 to induce joint instability. Because of the focal nature of the damage seen in this model [27], the region of the joint most affected (lateral or medial side) is dependent on the placement of the initial injection (that is, from which side). This placement varied between experiments but for individual experiments was kept constant. Mice were sacrificed at weeks 1, 2 and 6 post collagenase injection and knee joints were collected for histology.

### Pain readings

As an indicator of knee pain, the differential distribution of weight on the hind limbs was measured using an incapacitance meter (IITC Life Science Inc., Woodland Hills, CA, USA). This validated technique for arthritic knee pain [24,28,29] measures changes in weight distribution between the arthritic hind limb relative to the non-arthritic hind limb. Mice were allowed to acclimatize to the equipment on three occasions prior to experiment. Weight placed on each limb was measured over a 5-second period. Three separate measurements were taken per mouse for each time point by an independent observer without knowledge of the experimental groups. The readings were then averaged. Results are expressed as a percentage of the weight placed on the arthritic limb verses the contralateral control limb (arthritic limb/control limb × 100). A decrease in weight applied to the arthritic limb (that is, a reading <100) indicates pain [24].

### Antibody treatment

Mice with collagenase-induced arthritis were treated by intraperitoneal injection, either prophylactically or therapeutically, with anti-mouse GM-CSF monoclonal antibody (mAb; MP1-22E9, AbD Serotec, Oxford, UK) or IgG2a isotype control mAb (AbD Serotec) [7,10]. For prophylactic treatment, mice were given 250 µg per mouse per treatment of anti-GM-CSF or control mAb beginning 4 days prior to the first collagenase injection (day 0), with subsequent treatment on days -2 and 0, and thereafter three times per week for 6 weeks. Serum samples were collected at 2, 4 and 6 weeks post collagenase injection and investigated for their anti-GM-CSF mAb concentration. High serum levels of MP1-22E9 were detected at each time point, which, in the light of published potency data on MP1-22E9 [30], prompted us to reduce the dose used and extend the dosing intervals for the therapeutic treatment. For therapeutic treatment, mice were given 150 µg per mouse per treatment of anti-GM-CSF or control mAb twice per week following the onset of pain, until the end of the experiment at 6 weeks. The onset of pain was defined as a significant difference in the average pain compared with t = 0.

### Histology

At termination after arthritis induction, the knee joints were removed, fixed, decalcified, and paraffin embedded, as previously described [7,10]. Frontal sections (5 µm) were stained with either H & E to examine joint architecture or with safranin O, fast green and hematoxylin for proteoglycan loss, and evaluated by two independent observers without knowledge of the experimental groups, using the histologic assessment as follows.

Week 1 and 2 sections were scored for cellular infiltration and synovial hyperplasia from 0 (normal) to 3 (severe) as previously [21]. Week 6 sections were scored for cartilage damage in terms of the OA depth into cartilage (grade) and amount of cartilage affected (stage), as described by van Lent *et al*. [31]. This scoring system is modified from that of Pritzker *et al*. [32] to make it more suitable for measuring pathologic changes in murine cartilage. OA grade was scored from 0 (normal) to 6 (bone loss, remodeling, deformation) and OA stage from 0 (<10% involvement) to 4 (>50% involvement) for the lateral tibia, lateral femur, medial tibia, and medial femur. The grade and stage were then multiplied to give an overall OA score, to represent a combined assessment of OA severity and extent [31]. Up to six sections were scored per mouse, and an average taken. Safranin O-stained sections were used to assess the presence of osteophytes at the lateral and medial femur and tibia. Their size was analyzed using the image software Image J (National Institutes of Health, Bethesda, MD, USA ). The mean area per knee joint (three sections) was calculated and expressed in arbitrary units.

### Immunohistochemistry

The matrix metalloproteinase (MMP)-induced neoepitope, DIPEN, was detected as published [33]. Briefly, paraffin-embedded sections were deparaffinized and rehydrated, and endogenous peroxidise blocked with 3% (vol/vol) H_2_O_2 _(Sigma-Aldrich). Sections were digested with chondroitinase ABC (0.1 units/mL; Sigma-Aldrich) for 2 hours at 37°C to remove chondroitin sulfate from the proteoglycans, prior to blocking with 5% goat serum. Sections were then incubated overnight at 4°C with anti-DIPEN [34] (a gift from A/Professor Amanda Fosang, University of Melbourne, Victoria, Australia) and detected with a biotinylated anti-rabbit IgG (Dako, Glostrup, Denmark), followed by a streptavidin-peroxidase conjugate (BD Biosciences, San Jose, CA, USA). Peroxidase activity was demonstrated by incubation with 3,3'-diaminobenzidine/tetrahydrochloride (Sigma-Aldrich)-H_2_O_2 _solution. Slides were counterstained with hematoxylin.

DIPEN staining was scored from 0 to 3 based on amount of staining, where 0 = no staining and 3 = maximal staining, as previously described [35].

### Statistics

For pain readings, Student's t-test or two-way analysis of variance was used. For histologic scores, the Mann-Whitney two-sample rank test or two-way analysis of variance was used; values are expressed as the mean ± standard error of the mean. P * 0.05 was considered statistically significant.

## Results

### GM-CSF is important throughout collagenase-induced arthritis progression

**Figure 1:**
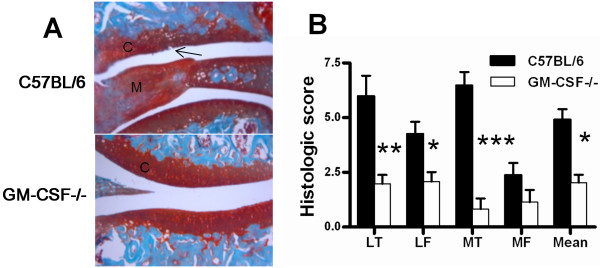
**GM-CSF is required for optimal expression of collagenase-induced arthritis**. Arthritis was initiated in C57BL/6 and GM-CSF-/- mice by intra-articular collagenase injection (Methods) and histology performed 6 weeks later. **(A) **Representative histologic pictures of knee joints (Safranin O/fast green stain) (original magnification ×100). **(B) **Quantification of arthritis development (histology) at day 42 (Methods). Results are expressed as the mean ± standard error of the mean; n = 10 mice per strain (from two independent experiments). *P < 0.05, **P < 0.01, ***P < 0.001, GM-CSF-/- versus C57BL/6 mice. C: cartilage; GM-CSF: granulocyte-macrophage colony-stimulating factor; LF: lateral femur; LT: lateral tibia; M: meniscus; MF: medial femur; MT: medial tibia; arrow indicates cartilage damage.

Several RA-like inflammatory arthritis models have been shown to be GM-CSF dependent [7-10]. To determine whether an OA-like model was also dependent on GM-CSF, histology was performed on the joints of C57BL/6 (wild-type) and GM-CSF-/- mice at 6 weeks post collagenase intra-articular injection and disease scored according to the published protocol [31]. At this time point, synovitis was not evident (data not shown). GM-CSF-/- mice showed less knee joint tissue damage in the lateral tibia (*P = *0.003), lateral femur (*P = *0.02) and medial tibia (*P = *0.001) compared with wild-type mice (Figure 1A,B). Overall the mean arthritis score for all regions was significantly lower in GM-CSF-/- mice compared with that in wild-type mice (*P = *0.002) (Figure 1B).

**Figure 2:**
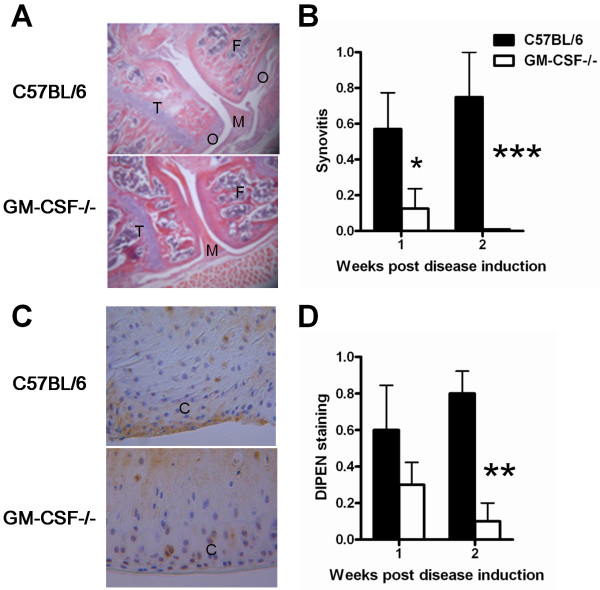
**GM-CSF is required for optimal synovitis and cartilage breakdown associated with collagenase-induced arthritis**. Arthritis was initiated in C57BL/6 and GM-CSF-/- mice by intra-articular collagenase injection and histology performed 1 and 2 weeks later. **(A) **Representative histologic pictures of knee joints at 2 weeks (original magnification ×100). **(B) **Quantification of synovitis. **(C) **Representative DIPEN staining in knee joints at 2 weeks (original magnification ×125). **(D) **Quantification of DIPEN staining. Results are expressed as the mean ± standard error of the mean; n = 5 to 8 mice per strain per time point (from two independent experiments).*P = 0.05, **P = 0.02, ***P = 0.002, GM-CSF-/- versus C57BL/6 mice. C: cartilage; F: femur; GM-CSF: granulocyte-macrophage colony-stimulating factor M: meniscus; O: osteophyte; T: tibia.

Synovitis and osteophyte formation have previously been reported to be present early in disease development in this model [21]. Therefore, a role for GM-CSF in the development of these disease features was examined at 1 and 2 weeks post collagenase injection. Mild synovitis was observed in wild-type mice at both time points (Figure 2A,B); however, this was virtually absent in GM-CSF-/- mice with only one mouse at week 1 having a very low score. Expression of the MMP-generated proteoglycan neoepitope, DIPEN, was assessed by immunohistochemistry as a measure of MMP-mediated cartilage damage [36]. At week 2, there was significantly greater expression of the neoepitope in the cartilage of wild-type mice compared with that in GM-CSF-/- mice (*P *= 0.02; Figure 2C,D). At both 1 and 2 weeks, there was a trend towards there being fewer osteophytes in GM-CSF-/- mice compared with wild-type mice, and a trend towards the mean osteophyte size being smaller in the former strain (data not shown). Therefore, GM-CSF plays a role throughout the arthritis progression in this model.

### Collagenase-induced arthritis pain is GM-CSF dependent

**Figure 3:**
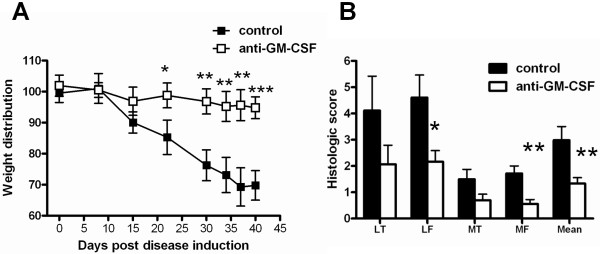
**Pain in collagenase-induced arthritis is GM-CSF dependent**. C57BL/6 mice were treated prophylactically (from day -4) with anti-GM-CSF or isotype control monoclonal antibody. **(A) **Change in weight distribution (pain) (Methods) over time. **(B) **Quantification of arthritis development (histology) at day 42. Results are expressed as the mean ± standard error of the mean; n = 10 to 15 mice per group (from two independent experiments). *P <0.05, **P <0.01, ***P <0.001, control versus anti-GM-CSF. GM-CSF: granulocyte-macrophage colony-stimulating factor; LF: lateral femur; LT: lateral tibia; MF: medial femur; MT: medial tibia.

We have recently shown that GM-CSF is key to the development of arthritic pain in a number of inflammatory arthritis models [24]. We next explored whether GM-CSF was critical also for joint pain development in this OA-like model by administering a neutralizing mAb to GM-CSF prophylactically. Pain has not been recorded in this model. Pain was measured by the changes in weight distribution using an incapacitance meter and has been validated previously to measure arthritic knee pain [24,28,29]. To assess whether there is pain induction, C57BL/6 mice received control mAb beginning 4 days prior to collagenase injection. Knee pain was evident from around 3 weeks and remained significant until 6 weeks when the mice were sacrificed (Figure 3A). Mice pretreated with anti-GM-CSF mAb, however, did not develop any detectable pain. From around day 22, control mAb-treated mice showed significantly more pain compared with anti-GM-CSF mAb-treated mice (*P *< 0.05).

In agreement with the GM-CSF-/- mouse arthritis data above, at 6 weeks the anti-GM-CSF mAb-treated mice also had significantly less arthritis in the lateral femur (*P = *0.03) and medial femur (*P = *0.004), compared with the control mAb-treated group (Figure 3B). To confirm that the isotype control mAb had no effect on disease development, a PBS control group was also included. There was no significant difference in the histologic scores for the PBS-treated group and the control mAb-treated group for any of the regions scored (data not shown).The mean level of arthritis development was significantly lower in the anti-GM-CSF mAb-treated group (*P = *0.003). Once again, synovitis could not be detected at this time point in any of the mice, and in mice where osteophytes were detected, they were significantly smaller in the anti-GM-CSF mAb-treated group compared with the control mAb-treated group (mean size: anti-GM-CSF mAb-treated group, 238 ± 114 versus control mAb-treated group, 577 ± 48, *P *< 0.05).

Thus GM-CSF is an essential mediator of knee pain progression in this OA-like model.

### Therapeutic neutralization of GM-CSF ameliorates both collagenase-induced arthritic pain and disease development

**Figure 4:**
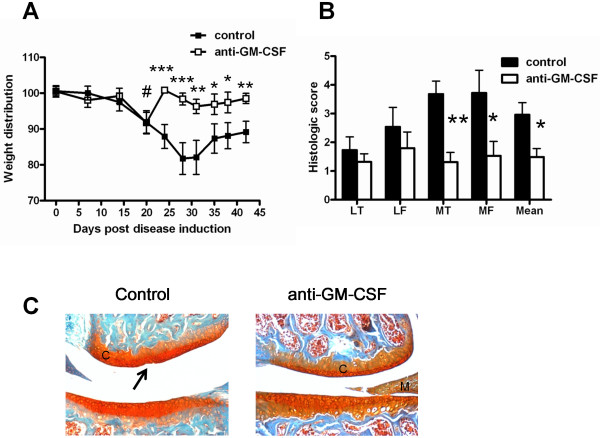
**Therapeutic neutralization of GM-CSF reverses collagenase-induced arthritis pain and disease**. C57BL/6 mice were treated therapeutically (from day 20) with either anti-GM-CSF or isotype control monoclonal antibody. **(A) **Change in weight distribution (pain) over time. **(B) **Quantification of arthritis development (histology) at day 42. **(C) **Representative histologic pictures of knee joints (safranin O/fast green stain) (original magnification ×100), arrow indicates cartilage damage. Results are expressed as the mean ± standard error of the mean; n = 10 to 15 mice per group (from two independent experiments). *P <0.05, **P <0.01, ***P <0.001, control versus anti-GM-CSF; # *P<*0.0001, control and anti-GM-CSF versus t = 0 readings. C: cartilage; GM-CSF: granulocyte-macrophage colony-stimulating factor; LF: lateral femur; LT: lateral tibia; M: meniscus; MF: medial femur; MT: medial tibia.

The mAb-based approach allowed us to next determine whether therapeutic treatment with anti-GM-CSF mAb could also suppress the pain and disease, and how rapidly it might reverse the pain. Once knee pain was evident in collagenase-injected C57BL/6 mice, those with similar pain readings were treated with either control mAb or anti-GM-CSF mAb twice weekly until week 6. Treatment with the control mAb had no effect on the pain, which continued to increase (Figure 4A). Treatment with anti-GM-CSF mAb completely suppressed the pain within 3 days, this being the earliest time at which it was measured following mAb treatment. This abolition of knee pain was maintained until sacrifice (day 42). By histology, at 6 weeks the anti-GM-CSF mAb-treated mice also had significantly milder disease in the medial tibia (*P *< 0.01) and medial femur (*P *< 0.05) regions of the joints (Figure 4B,C) with the mean level of arthritis development also being significantly lower in the anti-GM-CSF mAb-treated group for this therapeutic treatment protocol. Once again, in mice where osteophytes were detected, they were significantly smaller following anti-GM-CSF mAb treatment as compared with control mAb treatment (mean size: 267 ± 17 versus 406 ± 14, *P *< 0.05).

## Discussion

We demonstrated above for the first time that GM-CSF is required for both pain and optimal disease development in a joint instability OA model. Furthermore, GM-CSF neutralization by a therapeutic mAb-based protocol rapidly and completely abolished existing arthritic pain. Experimental OA, induced by intra-articular collagenase injection as above, has been shown to share some important features with human OA, such as the development of osteophytes and cartilage erosion [20,21]. There is also a low grade inflammatory response (synovitis), which can also be seen in the human disease [37]. The mild synovitis observed early in disease development was virtually absent when GM-CSF was not present. This is in agreement with previous studies using systemic inflammatory models of arthritis in which blockade or absence of GM-CSF led to a reduced inflammatory response and fewer macrophages in the joint [8,10]. Synovial macrophages have been shown to be important in this OA-like model, with both synovial inflammation and cartilage damage being ameliorated when they are selectively depleted from the synovial lining layer by clodronate liposomes [21]. As GM-CSF can directly promote macrophage survival [38], its neutralization may also lead to direct depletion of joint macrophages, in this case by apoptosis [38]. In the current study, the degree of MMP-mediated cartilage damage was also lower in the absence of GM-CSF, suggesting that GM-CSF may be important for sustained and ongoing joint damage, possibly by limiting synovial macrophage activation in addition to their numbers [2,4,38].

Osteophyte formation, which is a common feature of OA and which may be a repair mechanism to help stabilize joints [39], is prevented by synovial macrophage depletion in the collagenase-induced model [21]. An association between osteophytes and pain has been reported for hand OA [40]. In the absence of GM-CSF, the size of the osteophytes formed was smaller than in the presence of GM-CSF. This may be related to the lack of pain seen in the absence of GM-CSF. It could also indicate some requirement of (GM-CSF dependent) synovitis for osteophyte development, as has been previously suggested [41]; however, given there was still some osteophyte formation present in GM-CSF-/- mice, other factors may also be important, such as transforming growth factor β [42]. The degree of cartilage damage seen at 6 weeks post collagenase injection in the absence of GM-CSF was significantly decreased compared with that in wild-type mice. Interestingly, antibody-mediated GM-CSF blockade both prevented (prophylactic treatment) and reversed completely (therapeutic treatment) the arthritic pain and modulated the degree of osteoarthritic changes in the joint, indicating that GM-CSF is important both in the early and late stages in this model, even though the features of the model change over time. Of note, the neutralization of GM-CSF abolished existing pain within 3 days, the earliest time point measured. The effectiveness of the therapeutic protocol was similar to the findings for disease progression in systemic inflammatory and autoimmune models [7,10]. In addition to its direct effects on macrophages, GM-CSF may also have direct effects on neuronal sensitization in the OA lesion, similar to that suggested by Schweizerhof *et al*. [43] for bone cancer pain. Knee pain was evident 3 weeks post collagenase injection. The synovitis seen early in disease development in wild-type mice thus did not lead to any detectable pain, that is, a difference in weight distribution, unlike in more severe inflammatory models of arthritis [24,28,29]. This observation fits with the mild nature of the inflammatory response in this model. It would be of interest to determine the degree of synovitis at 3 weeks post collagenase injection, at the time of detectable pain, and to determine the effect of GM-CSF blockade at this time point on the synovitis. Recently, van Lent *et al*. [31] showed that synovitis peaks at week 2 in this model, before we were able to detect pain, further suggesting that the synovitis alone is unlikely to account for the pain. Exactly how GM-CSF is acting at the different stages is currently being investigated.

For the collagenase-induced model, cartilage damage is also less in IL-1β-/- mice [44], suggesting a link between GM-CSF and IL-1. Such a link has been found before with the GM-CSF dependence of an IL-1β-induced inflammatory monoarthritis model [8,24], consistent with the concept of a 'colony-stimulating factor network' linking GM-CSF, IL-1, and other cytokines [2,4,45]. That such a link between IL-1 and GM-CSF may also be operating in the collagenase-induced arthritis model is supported by the fact that IL-1 can enhance MMP-mediated DIPEN neoepitope expression in cartilage [46], similar to our data above for GM-CSF and neoepitope expression.

## Conclusions

From the above findings, it is proposed that GM-CSF is a key mediator of both pain and disease development in an experimental model of OA, in addition to its known role(s) in RA models. Both prophylactic and therapeutic blockade of GM-CSF were effective at ameliorating both pain and disease. Results from clinical trials assessing the role of GM-CSF in RA are encouraging [11,12]. Our results here suggest that it would be worth exploring the importance of GM-CSF for the pain and disease in other OA models and perhaps clinically for this form of arthritis.

## Abbreviations

GM-CSF: granulocyte-macrophage colony-stimulating factor; H & E: hematoxylin and eosin; IL: interleukin; mAb: monoclonal antibody; MMP: matrix metalloproteinase; OA: osteoarthritis; PBS: phosphate-buffered saline; RA: rheumatoid arthritis; TNF: tumor necrosis factor.

## Competing interests

SS is a full-time employee of MorphoSys AG, Germany. JAH has received consulting fees from MorphoSys AG, Germany (less than $10,000 a year). MD was a full-time employee of MorphoSys AG, Germany, until 2011. MorphoSys AG, Germany, have partially funded the work in this manuscript. Patent applications from the University of Melbourne (ADC, JAH) are pending on the treatment of OA and pain by using GM-CSF antagonists. MorphoSys AG hold patents on anti-GM-CSF antibodies.

## Authors' contributions

ADC conceived the study, and participated in its design and coordination, carried out the pain readings, and drafted the manuscript. SS and MD participated in the design of the study. JP, ELB, and ALT carried out the OA experiments and histology. DCL carried out the immunohistochemistry. JAH conceived the study and participated in its design and helped draft the manuscript. All authors read and approved the final manuscript.

## Acknowledgements

We thank Jennifer Davis and Lara Mizhiritsky for assistance with the maintenance and care of the mice, and Amanda J Fosang for the antibody against DIPEN. This work was supported by grants from MorphoSys AG and the National Health and Medical Research Council (NHMRC), and by a NHMRC Senior Principal Research Fellowship (JAH).
